# Lessons and Reflections From an Extended Co-design Process Developing an mHealth App With and for Older Adults: Multiphase, Mixed Methods Study

**DOI:** 10.2196/39189

**Published:** 2022-10-28

**Authors:** Catherine Tong, Alison Kernoghan, Kassandra Lemmon, Paige Fernandes, Jacobi Elliott, Veronica Sacco, Sheila Bodemer, Paul Stolee

**Affiliations:** 1 School of Public Health Sciences University of Waterloo Waterloo, ON Canada; 2 Lawson Health Research Institute London, ON Canada

**Keywords:** mobile health, mHealth, older adults, health care providers, co-design, user experience or UX design, qualitative, apps, elderly, health care, care provider

## Abstract

**Background:**

There are many mobile health (mHealth) apps for older adult patients, but research has found that broadly speaking, mHealth still fails to meet the specific needs of older adult users. Others have highlighted the need to embed users in the mHealth design process in a fulsome and meaningful way. Co-design has been widely used in the development of mHealth apps and involves stakeholders in each phase of the design and development process. The involvement of older adults in the co-design processes is variable. To date, co-design approaches have tended toward embedding the stakeholders in early phases (eg, predesign and generative) but not throughout.

**Objective:**

The aim of this study was to reflect on the processes and lessons learned from engaging in an extended co-design process to develop an mHealth app for older adults, with older users contributing at each phase. This study aimed to design an mHealth tool to assist older adults in coordinating their care with health care professionals and caregivers.

**Methods:**

Our work to conceptualize, develop, and test the mHealth app consisted of 4 phases: phase 1, consulting stakeholders; phase 2, app development and co-designing with older adults; phase 3, field-testing with a smaller sample of older adult volunteer testers; and phase 4, reflecting, internally, on lessons learned from this process. In each phase, we drew on qualitative methods, including in-depth interviews and focus groups, all of which were analyzed in NVivo 11, using team-based thematic analysis.

**Results:**

In phase 1, we identified key features that older adults and primary care providers wanted in an app, and each user group identified different priority features (older adults principally sought support to use the mHealth app, whereas primary care providers prioritized recoding illnesses, immunizations, and appointments). Phases 2 and 3 revealed significant mismatches between what the older adult users wanted and what our developers were able and willing to deliver. We were unable to craft the app that our consultations recommended, which the older adult field testers asked for. In phase 4, we reflected on our abilities to embed the voices and perspectives of older adults throughout the project when working with a developer not familiar with or committed to the core principles of co-design. We draw on this challenging experience to highlight several recommendations for those embarking on a co-design process that includes developers and IT vendors, researchers, and older adult users.

**Conclusions:**

Although our final mHealth app did not reflect all the needs and wishes of our older adult testers, our consultation process identified key features and contextual information essential for those developing apps to support older adults in managing their health and health care.

## Introduction

### Background

There is a growing interest in the role of technology in supporting older adults’ health and well-being. Emerging technologies may help older adults monitor their health by facilitating coordination of care, communication with members of their care team (including health care professionals and family caregivers), and self-management [1-3]. Mobile health (mHealth) technologies can improve patient experience [4], and enhance the delivery of health care by improving communication and collaboration and supporting health care professionals [3]. As Cameron et al [5] argue, “mobility is central to the notion of participatory healthcare,” allowing patients to engage in health care unconstrained (or less constrained) by time and space. While there are many definitions of mHealth [5], which overlap with eHealth, here we draw on the World Health Organizations description and distinction: mHealth is “the use of mobile wireless technologies for public health, or mHealth, is an integral part of eHealth, which refers to the cost-effective and secure use of information and communication technologies in support of health and health-related fields” [6].

The boundaries of mHealth have expanded rapidly with technological advancements and an increasing trend of accessing the internet through mobile and handheld tablet devices [7,8]. Despite the broad willingness of individuals to use mHealth technology to manage their health [9], research and product design in this field have been predominantly directed at one cohort of users: younger people [10,11]. Recent findings are challenging traditional stereotypes that suggest that older adults are afraid of, or unwilling to use, technology; older adults’ use of technologies, such as computers, mobile phones, tablets, and smartphones, has been steadily increasing in the past decade [12-14]. In addition, the COVID-19 pandemic has further accelerated older adults’ adoption of new technologies [15,16]. Older adults increasingly recognize that technology can play a role in supporting self-management practices through health monitoring and access to information [17-20].

There is emerging evidence specific to older patients, that mHealth tools could be adopted to support pain management [21], increase mobility (eg, [22]), mitigate fall risk [23], support healthy habits (eg, [24]), and manage a range of chronic conditions (eg, [25]). Many mHealth apps have been designed to support chronic disease management [26], including in older adult patients [3]. The development of mHealth apps has tended to echo the health care system that deals with specific diseases, conditions, and health care goals separately. What is lacking is a comprehensive mHealth app that would support an integrated approach to managing an older patient’s individual health care goals, needs, appointments, medication reminders, and health care communications.

Although some have questioned the clinical value of mHealth, because of a lack of evidence demonstrating broad impacts on patients [27], the sector has “exploded” [5]. There are many mHealth apps for older adult patients, but Wildenbos et al [28] scoping review found that broadly speaking, mHealth still fails to meet the specific needs of older adult users. According to Wildenbos et al [28], the development of mHealth apps for older adult users must consider cognition, motivation, physical ability, and perception, and be specifically mindful of physical and perceptual barriers. These considerations have also been echoed by Li et al [29] in their reflections on mHealth apps for older users.

### Co-design and User Experience With Older Adults

The comments from Wildenbos et al [28] and Li et al [29] highlight the need to embed users in the mHealth design process in a fulsome and meaningful way. The principle of designing with the user, for the user, is reflected in both a “co-design” approach and user experience (UX) approach. Co-design has been widely used in the development of mHealth apps (see [30]) and involves stakeholders in each phase of the design and development process. The involvement of older adults in the co-design processes is variable [31]. The systematic review by Noorbergen [30] found that co-design approaches tend to embed stakeholders in early phases (eg, predesign and generative) but not throughout. Conceptually similar to co-design, UX or UX design emphasizes incorporating the perceptions of users resulting from their own experiences using a service or product through processes such as usability testing [32,33]. Within the UX field, principles of human-centered design can support the development of products that address the needs and capabilities of users [34]. Focusing on the needs and capabilities of users also reflects what Wildenboss et al [28] and Li et al [29] specifically stated about designing for older users. More recently, researchers have offered specific UX design approaches with and for older adults, notably Russell Kirkscey [33,35]. Relationships and trust building have been identified as important elements of these approaches [31]. This growing body of literature suggests that without embedding the user throughout, without focusing on the needs and capabilities of users, or without relationship and trust building between users and developers, co-design may not produce the intended outcomes.

The aim of this study was to reflect on the processes and lessons learned from engaging in an extended co-design process to develop an mHealth app for older adults. The aim was to design an mHealth tool to assist older adults in coordinating their care with health care professionals and caregivers, with an emphasis on primary care. Our research question and objective for this manuscript are as follows:

- Research question: What do older adults, primary care providers, and other stakeholders wish to see in an mHealth app that supports older patients in managing their health and health care?
- Manuscript objective: What did we learn from an extended co-design process involving older adults and developers? How may our reflections and lessons learned inform our future work and those of others?

This project was nested within a broader, multicomponent intervention designed to transform primary care for older patients and their caregivers in 3 Canadian provinces [36]. The process for informing, developing, and testing the app is presented below.

## Methods

### Overview

Our work to conceptualize, develop, and test the mHealth app consisted of 4 phases: phase 1: consulting stakeholders (Jan-Nov, 2017); phase 2: app development and co-designing with older adults (Jan-Sept, 2018); phase 3: field-testing with a smaller sample of older adult volunteer testers (Jan-April, 2019); and phase 4, reflecting, internally, on lessons learned from this process (Sept-Dec, 2020). The methods used to track, report, and understand each phase are outlined below. We are a team of mixed methods researchers, gerontologists, and professionals in the geriatric health care sector who came together to implement a multifaceted intervention to improve primary care for older patients. This was the first app development initiative undertaken by our team.

### Phase 1 Methods: Consulting Stakeholders, After a Scoping Review

Before phase 1, we conducted a scoping review of the types of mHealth tools that exist to support care coordination for older adults living in the community, as well as their existing and desired features and implementation issues (results reported elsewhere; [37]). Findings from the scoping review informed a consultation process with stakeholders, as per the scoping review methodology by Levac et al [36]. Through the consultation step and alignment with the principles of a co-design approach [30], we aimed to better understand the mHealth preferences of key stakeholders, including older adults, family caregivers, and primary care providers.

A total of 26 participants were recruited from both urban and rural locations in Southern Ontario, Canada. Data collection included individual interviews with 5 primary care providers, 1 caregiver, and 1 technology expert, and 4 focus group interviews (4-6 participants each) with older adults and caregivers. Older adults were defined as persons aged ≥65 years who were living in the community accessing primary care services and who were able to speak English and provide their own consent. Family caregivers were persons of any age who took the role of caring for older adults living in the community. Providers included persons of any age who played the role of primary care providers, such as family physicians or nurse practitioners. As this work was embedded in a larger project aimed at transforming primary care for older patients, we leveraged existing partnerships with 5 clinics and relevant organizations (eg, patient advisory groups) to recruit, using both a recruitment script (delivered by gatekeepers in each group) and recruitment posters. Participants were interviewed (either individually or via a focus group) and asked to complete a priority-setting questionnaire, where they ranked mHealth app features, which had been derived from the scoping review, in order of importance. The interview guides are presented in Multimedia Appendix 1, and the questionnaires in Multimedia Appendix 2.

Semistructured interviews and focus groups with participants were used to obtain a richer understanding of mHealth’s preferences. These individual and focus group interviews lasted approximately 45-60 minutes and were digitally recorded and transcribed. Interview data were analyzed by 1 graduate student (PF) using the thematic analysis approach by Braun and Clarke [38], supported by NVivo (Version 11, QSR International). Data from the questionnaire were entered into an Excel spreadsheet, averaged, and ranked based on the mean ratings. SDs were calculated based on sample variance and reported as indicators of consensus. The highest rated features were considered as priorities to be included in an app.

### Phase 2 Methods: App Development and Co-designing With Older Adults

After completing the background scoping review and consulting with stakeholders to understand what should go into an app to support older patients and their caregivers, particularly in primary care, we began the app development and co-design process. Our research team led co-design sessions with older adults, and we contracted local developers to create the coding required for the app. App development and consultations with older adults occurred simultaneously, with the intent of using older adult feedback at each stage of the development process. Older adults were recruited from the Seniors Helping as Research Partners (SHARP) group, with which our team had worked for more than 7 years, and who were also involved in the interviews described in phase 1. During the app development process, we conducted 3 in-person co-design sessions with approximately 6 older adults per session, led by team members trained in facilitation and qualitative data collection. We communicated the findings of the older adult co-design sessions via team meetings with developers and more than 100 emails. To report and reflect on this process, we have drawn on the development contracts and letters of support, field notes from the co-design sessions, and minutes and recordings from 15 meetings with the developers, whom we have anonymized here.

### Phase 3 Methods: Field-testing With Older Adults

Participants who had been involved in the earlier stages of the co-design process were contacted by researchers via email to invite their participation in testing the app. A total of 6 participants (5 older adults and 1 caregiver) from a midsized city in Southern Ontario agreed to field-test the app for a 6-month period. Interested participants completed individual, in-person training tutorials (20-30 minutes in length) with a research associate. During one-on-one training sessions, research associates assisted participants with logging into the app using an iPad or Samsung tablet provided by the research team to ensure that testing was completed in both Apple (iPad) and Android (Samsung) formats. The researchers guided participants in a step-by-step tutorial on how to use each of the features available within the app. An Apple- or Android-specific user guide for the app was provided to each participant to provide basic information on how to use the tablet device and app. A research assistant connected individually with each field tester at the 2-week mark, and then monthly. Communication with the field testers depended on personal preference and included telephone calls, in-person meetings, and emails with the research team. Field testers were asked to take notes on specific features in the app; these note-taking templates are available in Multimedia Appendix 1. Scheduled conversations with field testers were digitally recorded and guided by the interview questions listed in Multimedia Appendix 1.

### Phase 4 Methods: Reflecting on Lessons Learned

To explore our team’s reflections and lessons learned from the process, a small focus group session (n=3) was held with members of the team closely involved with the co-design project from its inception. We recorded and transcribed the focus group and applied thematic analysis [38] to identify the lessons learned.

### Ethics Approval

We received ethics clearance from the University of Waterloo’s Office of Research Ethics (ORE # 44428) for phases 1, 2, and 3; phase 4 only included members of the research team and all coauthors who agreed to share and record their reflections for this manuscript.

## Results

In the following sections, we highlight the findings for each phase. In phase 1, we identify the mHealth features that older adults and primary care providers value and contextual considerations from interviews and focus groups; in phase 2, we share observations from the app development and co-design process; in phase 3, we briefly describe field-testing; and in phase 4, we describe the lessons learned from this co-design process, drawing on a reflective focus group with our research team.

### Phase 1 Findings: Consulting Stakeholders

In the questionnaires, older adults and caregivers reported that they were most interested in training and supporting the use of the tool, keeping a contact list with their care team, reminders to bring items to appointments, and the ability to track their illnesses. Meanwhile, primary care providers were interested in older adults bringing their medications, appointment details, contact list of the older patient’s care team, goal setting, tracking exercise, alerts if their health data were out of range, and reminders to track health information. Table 1 displays the top 10 mHealth feature priorities of older adults, caregivers, and primary care providers identified in the questionnaires.

Textbox 1 outlines the 8 major themes identified in our analysis of the interviews with stakeholders. While the surveys identified desirable features and support for an mHealth app, the interviews revealed and expanded on important contextual factors that could support or impede the use of such an app. These contextual factors were connected to the broader (disjointed) health care system where we work, access to technology (eg, limitations in more rural areas), and more individual-level considerations, including individual patient and caregiver differences and preferences, access and comfort with technology, and health literacy. For example:

Dr X has something, another doctor has something, your specialist has something, another specialist got something, another bone specialist has got something, the (police) has something, the fire department’s got something from my wife. Everybody got something, but what do you do with it all?

caregiver 2

...and there are some not so good EMRs and you couldn’t interface with anything.

Health care provider 1

Participants also discussed the utility and potential of mHealth apps and generally felt very positive about mHealth; however, important considerations may limit uptake or reproduce existing health inequalities (eg, for people with limited resources, low health literacy, very poor health). Participants also emphasized that an mHealth app would likely be more successful if it was offered and aimed at caregivers supporting frail older adults:

A lot of my patients have great caregivers, daughters, sons who come to appointments with them. They would be more likely to adopt the app like that and keep it up to date and they have their own busy life.

Health care provider 4

Consultation processes with key stakeholders confirmed that older adults and primary care providers have a strong interest in mHealth tools and pointed to features that should be integrated into an mHealth tool to support care coordination. This background work laid the foundation for the next phases of the project, including partnering with an app developer to create an mHealth tool and testing older adults and family caregivers.

**Table 1:** Mobile health feature priorities from consultation questionnaire.

Rank^a^	Health care provider	Value, mean (SD)	Older adult or caregiver	Value, mean (SD)
1	My immunization records	5.00 (0.00)	I will be able to call a telephone support line if I need help using the app or setting it up	4.44 (1.34)
2	My illnesses	5.00 (0.00)	I will be given a face-to-face training session on how to use the app	4.39 (1.42)
3	Appointment name (eg, Cardiologist appointment, Dr __)	5.00 (0.00)	I will be given a user manual with written instructions of how to use the app	4.28 (1.41)
4	I will be able to keep a contact list and information of all those involved in my care team (eg, physician, Nurse, Specialists, etc.)	5.00 (0.00)	Appointment name (eg, Cardiologist appointment, Dr _________)	4.22 (1.52)
5	My medication	4.8 (0.45)	There will be a tutorial within the app to explain to me how to set-up and use it	4.22 (1.44)
6	I will have the ability to give access to others (health care providers or caregivers)	4.8 (0.45)	Prepare for appointments—bring medications	4.16 (1.50)
7	Prepare for appointments—bring medications	4.8 (0.45)	Having the option of a paper-based or hard-copy version rather than web-based version	4.12 (1.27)
8	Appointment details—date and time	4.8 (0.45)	Prepare for appointments—bring health documentation	4.11 (1.56)
9	I will have the ability to track my symptoms	4.6 (0.55)	I will be able to keep a contact list and information of all those involved in my care team (eg, physician, Nurse, Specialists, etc.)	4.11 (1.66)
10	I will be able to create personal health goals	4.6 (0.55)	Appointment details—location	4.06 (1.70)

^a^These priorities were identified based on the mean averages from each question in the questionnaire (5-point scale, with 5 being very interested).

**Summary of themes and subthemes from qualitative consultation with key stakeholders.**

**System level gaps impact care coordination and self-management**

- Problems with information transfer between health care professionals or settings
- Lack of standardization in care coordination practices
- Lag periods between appointments
- Short appointment times with health care professionals
- Challenges with navigating the system

**Microlevel issues impact or prevent self-management**

- No standard tracking method
- Patients’ needs vary from simple to complicated conditions
- No equipment at home to monitor own health
- Lack of understanding of health conditions
- Provider does not provide all information to the patient or caregiver
- Caregiver feels burdened managing information

**Older adults currently self-manage their health in various ways**

- Tools patients use to keep track of their health information: spouse or caregiver; memory; diary or notebook; pill boxes; paper copies of documents

**Positive experiences empower older adult patients to self-manage health**

- Importance of self-advocacy to get information
- Understanding health status
- Building trust or relationships with patients and health care

**Technology can support self-management practice in various ways**

- Monitoring via devices
- Phone reminders
- Memo or notepad on phone
- Web-based laboratory results
- Phone calendar

**Apps or technology can support current practices for older adults and caregivers**

- Participants’ vision of using apps
- Suggested app features
- Suggested design esthetics

**Technology can be a barrier to adopting or accepting self-management practices**

- Limited access to technology
- Privacy concerns
- Financial barriers
- Negative attitude toward technology
- Age as a barrier
- Cognitive impairment
- Technology illiteracy
- Cultural differences
- Transition from paper to technology
- Negative attitudes to tracking health

**Considerations for implementing technologies for patients and health care professionals**

- Training education for the following: providers on the technology and how it is used; helping end users use the technology
- Developing an implementation strategy for patients and health care professionals
- Need for discussions to be had on what information patients need to track

### Phase 2 Findings: App Development and Co-designing With Older Adults

Our team is situated in the largest technology hub in Canada, and we relied on guidance from colleagues familiar with the local developer context to select an appropriate vendor when developing our research proposal for funding. We engaged in conversations with vendors in 2017 and worked with university staff (eg, our university privacy officer) before selecting a developer to understand issues related to privacy, given that the app would store personal health information for users. Given the nature of our research funding, we had to have vendors selected, with “letters of support” at the point of applying for funding, long before any funds were available, or details were finalized. We selected our vendor, given their prior experience in developing mHealth apps, who began work in early 2018.

Drawing on our consultation work, the app was intended to help store and organize older adults’ health care information, including the professionals and clinics involved in their care, appointments, and medication lists. The app also reminded users of upcoming appointments and tasks, in addition to allowing older adults to track and monitor health information, such as weight, blood pressure, or physical activity. Older adults from SHARP group reviewed paper mockups of the health app and provided preliminary feedback. SHARP members predominantly highlighted issues around accessibility, including small font size, use of colors, minimizing language complexity, and simplifying navigation. App developers implemented this initial feedback to create a web-based app prototype. Older adults from the SHARP group were asked to review the prototype and provide comments on the platform. Researchers and app developers incorporated some of this feedback into the app design before its launch on the App Store and Google Play Store, which are available in English and French.

The next step involved testing the app with group members of SHARP. Older adults in this group tested the app for approximately 2 weeks, on their own devices or a loaned device from our team, before providing additional comments to the research team. While users appreciated the general appearance of the app and app icon, some older adults found it cumbersome to navigate through the app and the accompanying manual. Older adults suggested providing additional training on how to use all the features on the app, along with providing styluses. Researchers and developers have attempted to incorporate this feedback into an update for the app before starting field-testing; however, many suggestions could not be implemented, as our vendor perceived these additional changes to be outside the scope of our agreements. For example, older adults wanted the option to view their calendar of appointments both weekly and monthly, but developers deemed this “out of scope.” Testers would also have appreciated the option to consolidate the calendar in the app with the existing calendar on their devices; likewise, this was deemed out of scope.

### Expectations and Challenges in Working With the Vendor

We contracted the vendor for 11 months. In the first 2 months, we regularly met with the vendor and concurrently engaged in focus groups with older adults (outlined above) to review the app development process. While we, as health researchers, called this “co-design” or “co-creation” [39,40], many in the development sector would have labeled this UX.

By month 4, we realized a mismatch between our expectations and what the vendor was willing or able to deliver. We provided feedback from the co-design process with older adults in months 2 and 3, but very little user feedback was implemented. Although our initial agreement noted that the vendor would “rely heavily on the user’s input,” and the contract stated the developer planned to “engage with the project team in an iterative fashion,” we viewed this engagement as quite limited. While some requests were justifiably out of scope (eg, linking the app to the existing electronic medical record [EMR] systems of the users’ physicians), many requests appeared (in our minds) relatively straightforward and were covered by our initial agreements. There were also occasions in what the older adults wanted (eg, specific ways to navigate the app or visual preferences) were not aligned with what the developers deemed “best practices” in their field (eg, which some users requested text to be in all caps for readability and vision issues, this is generally avoided in app development). This example, however, raises the question: When designing an app for older adults, should their preferences or “best practices” take precedence? The final product was not reflective of user input and feedback.

### Phase 3 Findings: Field-testing With Older Adults

After launching the app on Apple and Google Stores, we distributed the devices with the app (and styluses to support usability) to 6 volunteer field testers with the intention of eliciting their feedback and tracking their use of the app for 6 months (eg, to determine which features were being used most often, to understand if the app was taken to medical appointments or used in conjunction with caregivers, etc.). Unfortunately, 2 participants returned their devices before study commencement: one because they found the app too complex and the other because of visibility issues (they reported that they would have preferred all text in the app to be capitalized for better readability or a setting that allowed the user to change the font to capitalized, depending on one’s preference).

The initial results showed that the app supported the management of some aspects of participants’ health or health care. For example, one participant found the Reminder function in the “My Calendar” element to be especially helpful in organizing their health care (eg, appointment and medication reminders) and another found the Care Team feature to be particularly beneficial for consolidating the contact information of their health care professionals. However, the bulk of the feedback reflected that most inputs from older adults in phase 2 (development and co-design) were not addressed. There were consistent usability and accessibility issues (eg, small font size, readability, and overall complexity of the app) and features that were available but not functional in a way that the participants envisioned (eg, there was no mechanism to link the calendar in the app to the existing calendars on the devices, so participants had to maintain 2 calendars, one for their health in the app, and one on their device for regular scheduling of personal and professional events). Field-testing was halted after 3 months, given consistently poor feedback from the remaining testers. Although we launched this publicly, the app was not rolled out as part of our wider intervention in primary care, given the usability concerns raised above.

### Phase 4 Findings: Reflecting on Lessons Learned

#### Overview

Suspending field-testing and not rolling out the app for use on a wider scale was not the intended outcome, and our team concluded this work with a reflective focus group to try and better understand the lessons learned. The lessons are summarized in Table 2 and in the narrative below.

**Table 2:** Lessons learned in the development of an mHealth app with and for older adults.

Lessons learned	Description	Supporting quotations, from the research team
1. Selecting a strategic partnership with aligned goals	Ensuring that partnerships between research teams and tech developers are grounded in an understanding of each other’s goals and priorities and that the project will be mutually beneficial for both groups.	“We need to do our due diligence to go out and interview different teams...and select one that shares the same values and wants to work the same way that we have in mind.” [P 1]
2. Including a person on the research team with content expertise in tech development	Researchers commented on not speaking the same “language” as tech developers, and this posed some challenges around what was or was not possible as the project evolved. Incorporating a consultant with relevant experience in the technology and app development space was proposed as a suggestion.	“Have someone even in the know, review what the other company is saying...So maybe have someone look over their letter of support or what their proposal would be.” [P 3]
3. Facilitating direct relationships between users (ie, older adults) and tech developers from the beginning of the project	The research team can play a significant role in coordinating relationship-building between older adult users and app developers, commencing at the start of the project. As research teams may already have preexisting connections with older adults in the community, they can leverage these relationships to bridge the gap between users and tech developers to co-develop impactful products.	“And then during that meeting at the end of the day, functionally, what does an older adult want, and they can talk about that a lot. And then the tech people can sit there and sort of analyze what’s possible or not...if we brought these groups together from the beginning, and had more planning ahead of time, we might have started a little bit differently.” [P 2]

#### Selecting a Strategic Partnership With Aligned Goals

One of the most significant findings resulting from this project is ensuring that partnerships between research teams and technology developers are grounded in an understanding of each other’s goals and priorities and that the project will be mutually beneficial for both groups. Our research team felt this was not the case for our project, evidenced by the following member’s statement: “We’re going into a business agreement with another organization who doesn’t have the same end goals as we do with this product” [P 2].

Selecting these strategic partnerships can be a challenge for research teams to write grant proposals, requiring them to submit partnership letters and draft a budget under time constraints. However, building in time to meet with different vendors and determining which company’s values and approaches best align with those of the research team is an imperative step in supporting a productive partnership. Another difficulty we encountered was the different understandings of what is involved in a partnership. On the basis of our team’s personal experiences, the developer we worked with was rooted in a “business model” where strictly following the contract was prioritized. However, our understanding of a partnership involves more iterative and flexible processes, such as opportunities for ongoing feedback and making necessary adjustments to the app. When developing contracts with a vendor, researchers should ensure that their team wishes to engage in iterative processes, such as multiple rounds of feedback, which must be explicitly built into the contract from the outset.

#### Including a Person on the Research Team With Content Expertise in Tech Development

Researchers commented on not speaking the same “language” as tech developers, which created some tension around what was or was not possible as the project continued. Because of the iterative nature of the co-design processes, participants involved in testing the app provided feedback at multiple stages. Throughout this process, certain suggestions could not be actioned because the app developers felt that they extended beyond the project’s scope or were not aligned with “best practices” in the developer field. The research team agreed that some recommendations were beyond the project’s scope, such as “older adults really wanting a system that integrates in with their primary care EMRs…so that they could have conversations with physicians through the app” [P 1]. It would have also been helpful, particularly at the outset, to have a team member who understood both the length of time it takes to co-design an app and the longer-term commitments required to maintain the app. Finally, it is incredibly challenging to integrate an mHealth app into a health care system that itself is not integrated (eg, if a primary care provider and specialist do not use the same EMR system, how would an app for older adults integrate with these disparate systems?) However, our team felt that other concerns raised by older adults, especially pertaining to the app’s visual design and operability of features, were more feasible for technology developers to address. One member of the research team suggested incorporating a collaborator with relevant experience in the technology and app development space, which could help the team navigate any areas of contention as the project evolves.

#### Facilitating Direct Relationships Between Users (ie, Older Adults) and Tech Developers From the Beginning of the Project

The research team can play a significant role in coordinating relationship-building between older adult users and app developers, commencing at the start of the project. As research teams may already have pre-existing connections with older adults in the community, they can leverage these relationships to bridge the gap between users and technology developers to co-develop impactful products. In the context of our project, researchers took on the role of being the “middlemen” between older adult users and tech developers. We elicited feedback from older adults and relayed information back to tech developers; however, these 2 groups were never brought together, which posed significant limitations. This approach was inefficient because researchers were communicating separately with both groups and tech developers did not receive input directly from users to better appreciate each other’s perspectives. One of our team members highlighted this issue by saying, “If we brought these groups together from the beginning, and had more planning ahead of time, we might have started a little bit differently” [P 2].

## Discussion

### Principal Findings

Through our multiphase, mixed methods co-design project, we learned that (1) older adults and primary care providers are keen on an integrated app that helps older adult users manage their health and health care, (2) older adults prioritized multiple modes of support to use the app effectively, whereas primary care providers emphasized the importance of features that helped track (eg, track appointments, the care team, vaccines, etc), (3) co-designing with older adults and developers was fruitful, in terms of learning, but ultimately challenging. We also included a post hoc analysis of the oft-cited “pitfalls” [30] of mHealth development, with the intent to be reflective and inform the future work of our team and others. Both steps offer novel insights into mHealth development for older users. Implications for app development and practice, and implications for future UX and co-design research, are further detailed below.

### Implications for mHealth App Development and Practice

Our consultation phase identified the features, functions, and important considerations (eg, privacy, accessibility, and affordability) that older adults, caregivers, and primary care providers wish to see in mHealth apps designed to support the health and health care of older patients. Our findings specific to mHealth features and considerations are consistent with other research (eg, [3]) and highlight the importance of perceived usefulness and perceived ease of use [41]. Notably, primary care providers ranked immunization records and a list of illnesses as their top 2 features, whereas older adults identified IT user support and training to use the app as their 2 most highly ranked features. We sought to address these priorities and create an app that reflected the preferences of both primary care providers and intended users (ie, older adults). In our case, these preferences were not contradictory: the providers wanted specific features (eg, something to track appointments and vaccines), whereas the older adults wanted support tools to accompany the app (eg, manuals or IT support) so that they could use the features.

When working with the developers, we also saw differences between what the “experts” or professionals were recommending and what older users asked for (eg, asking for design elements that contradict best practices and design standards). In our experience, both users and developers have strong reasons for their preferences, and (in hindsight) we should have built more time into the initial contract for developers to work directly with older adults to come to a shared understanding of conflicting elements.

Some of the features that both primary care providers and older adults wanted were not possible in our health care system. It is fundamentally impossible to integrate an mHealth app into a broader, multifaceted health care system that is not itself integrated [42-44]. In a context in which primary care clinics, hospitals, specialists, and home and community care are potentially all using different EMR systems, or perhaps not using an EMR at all [45], we were not able to respond to the requests for features that connected patients to their records, health histories, appointments, etc, across numerous, disparate systems. While we were aware of the disconnected nature of our system, perhaps we did not fully appreciate the argument by Kirkscey [33] that “to find any measure of success, a fully functional app for older users should be integrated into the entire health-care system.” Our participants echoed Kirkscey in our consultation phase: the execution of an integrated app is limited in contexts in which the health care system itself has yet to integrate. For those working in a disconnected health care system, and with co-designing technologies, it may be helpful to provide the co-design participants with some context regarding what is (and what is likely not) possible in their particular setting and context.

### Comparison With Prior Work and Future Directions: UX Design

Unlike most UX approaches in the review by Noorbergen [30], we focused on including users in each step of the co-design process. Consistent with the UX literature [32,33,35,44], we aimed to address the perceptions, needs, and capabilities of our users; however, our IT vendor was not always able to address their needs and requests in the development process. Although UX researchers [31] have emphasized the importance of trust and relationships, in our experience, this was not lacking with the field testers but with the developers. Devoting time to relationships and trust building, including time for socializing, is recommended as a facilitator of the co-design process [31], but this is complicated when working with developers who must charge for the time they devote to the project.

As more health researchers attempt to leverage technology’s potential to improve the well-being of older adults [1], it is possible (in our experience) that researchers will confront unforeseen challenges in the tech development sector [30]. One aspect overlooked by our team is the importance of ensuring that researchers and app developers are committed to adopting a UX lens when working with user stakeholders to cocreate mHealth technologies for older adults [33,35]. Our study’s design broadly aligns with the aspects of human-centered design outlined by Harte et al [34], such as engaging in iterative processes and involving users throughout the different phases of the app’s design and development. Our work further adds to this conversation by showcasing challenges surrounding partnerships between researchers, app developers, and stakeholder users that research teams should consider [46] when co-designing an mHealth tool for older adults, even when following the appropriate guiding principles and standards of UX.

UX design has notably developed and evolved in the last few years, including the application of UX to the development of mHealth apps [33,47-49], and it is likely that teams engaged in this work will find it easier to find developers versed in the UX principles. There is also a broad body of literature on this topic. Much has been written about the barriers to mHealth usage by older adults (eg, [21,28,29]), and our findings from the consultation phase echo the many recommendations and considerations specific to visibility (eg, clear text, contrast), accessibility (eg, ability to zoom in, translate, change font size), and the importance of ease of use (eg, simple navigation, explicitly noting links). We have also seen the emergence of literature on how to design mHealth apps with and for older adults (eg, [29,33,35]), as well as insights and guidance on designing for older persons living with dementia [50], and individuals with a range of physical limitations [51]. This newer body of literature reflects our approach, which embeds older adults in every developmental phase. The limitations of our final product were not necessarily because of a faulty approach, per se, but rather a development partnership that did not prioritize or reflect what we were hearing in our engagement with older adults. UXs and feedback must be meaningfully adopted at each stage of the development process [29,30,33]; this can help ensure that all perspectives are considered and to avoid unrealistic expectations [31]. We would recommend that researchers, clinicians, and developers entering into the mHealth app development process take some time to ensure everyone is on the same page, not just technically but also in their approaches to UX or co-design, long before any contracts are developed.

### Agism Against Older Adults and Gerotechnology

The COVID-19 pandemic illuminated and reproduced agist (against older adults) attitudes that have permeated our society for decades; if not longer [52,53]. We live in an ageist society that prioritizes the experiences, preferences, wants, and needs of younger people [54]. Although we would never suggest that the developers that we worked with were explicitly agist, they are (just as we are) part of a society that prioritizes the needs of younger people and are also part of an industry that has historically done a better job designing for younger people [10,11]. We are encouraged to see guidelines and recommendations for designing apps for older users (eg, [29]) and the rising prominence of gerontechnology, a gerontological discipline dedicated to the design and adoption of new technologies for older people [55]. Although researchers have been engaged in gerontechnology for more than 20 years [56], our work suggests that more work is required. Our findings demonstrate a clear desire for an app (or apps) that supports patients in managing information about their appointments, care team, chronic conditions, prescriptions, vaccinations, etc. We are sharing these results in a time when the population is aging, older adults are living longer (but often with a higher number of chronic conditions and prescriptions to manage) [57], when (on account of the COVID-19 pandemic) keeping track of vaccinations has become even more important and older users have increasingly integrated technology and smartphones in their daily lives [15,16], In an ageist *and* aging society, there is both a moral imperative and strong business case to be made for designing for the older user.

### Strengths and Limitations

The review by Stowell et al [58] has shown that UX design has often overlooked the experiences and input of racialized users, and our sample of older adults was also predominantly White and of relatively high socioeconomic status (a description of the SHARP group can be found in [59]). Given the nature of our findings, this study also does not include the perspectives of the developers we worked with; it is likely that their interpretation of our process and the product delivered would be different. Teams engaging in a UX development process with an external partner may wish to proactively build a “postlaunch debrief” session into their initial contracts, knowing that these processes can be complex and merit a post hoc analysis of what did and did not work for both parties. Finally, although our final mHealth product has real limitations, we would also argue that peer-reviewed literature tends to focus on success stories, and there is great value in reporting on missteps and deviations to inform the work of others [60]. We situate some of our findings (namely phases 2 and 4) within broader calls for publishing “negative” results (eg, [61]), not just research success stories. We believe in the power and importance of designing with and for older adult users and have continued to do so across several high- and low-tech projects.

### Conclusions

Although our final mHealth app did not reflect all the needs and wishes of our older adult testers, our consultation process identified key features and contextual information essential for those developing apps to support older adults in managing their health and health care. Furthermore, our reflective process identified important factors to consider when health researchers and gerontologists enter the app development sector. In the words of Karl Popper [62], “every refutation should be regarded as a great success,” and we hope that the reflections, and refutations, shared here will inform and support the future work of others seeking to support the health of older adults using mHealth apps.

## Appendix

Multimedia Appendix 1 Interview guide and field-testing notes template.

Multimedia Appendix 2 Questionnaires.

## Abbreviations

EMR: electronic medical record
mHealth: mobile health
UX: user experience
SHARP: Seniors Helping as Research Partners
