# Supplementary material for: Lessons and Reflections From an Extended Co-design Process Developing an mHealth App With and for Older Adults: Multiphase, Mixed Methods Study
Source: JMIR Aging. 2022 Oct 28;5(4):e39189. doi: 10.2196/39189 (PMC9652733; doi:10.2196/39189)
Supplement: Multimedia Appendix 1 [file aging_v5i4e39189_app1.docx]

# Appendix A: Interview Guides

**Healthcare Provider Individual & Focus Group Guide**

Introduction Script (*accompanied by a Letter of Information & Consent Form*)

Thank you for taking the time to participate in this study. I want to remind you that you may choose not to answer any of the questions and can end your participation at any time during this interview. Your confidentiality will be respected and your name will not be associated with the data. With your permission, we will be audio-recording this session.

In our study, we are looking to find out whether older adults and their family members would benefit from using such an app, and what things older adults and family members may find useful. I am also looking for the perspective of healthcare providers on the development of an app to support patients and their family members.

1. What types of personal health information such as blood pressures, medications, etc. do you find is important for patients to keep track of when they come in for medical appointments?
2. How do you usually share information with your patients and how do your patients keep record of this?

*Probe: do they bring a notebook with them and write it down?*

1. Do you feel that an app on your smartphone or tablet would be helpful for your patients to keep track of the information that was discussed earlier? Why or why not?
2. What features do you think would make your patients want to use the app? What should it do for them?

*Probe: texting capabilities with a doctor, look at your own data, keep track of your own records, conversations, and appointments, reminders, service locator*

1. What are some concerns that you think would prevent your patients from using this type of app?

*Probe: older adults feel there is no use for technology? Privacy concerns? Low computer literacy? Not appropriate target population?*

1. What would prevent you from using or promoting the use of an app that helps older adult/patients keep track of their health information?

*Probe: having to learn how to use the tool? Feeling that the patient won’t understand how to use it?*

1. What are some gaps you see were not identified during this interview? Any additional comments or feedback?

At the end of the interview, I will hand the participants the feature preference questionnaire and give them 5-10 minutes to complete. If it is a phone interview I will ask them to complete this online and send back via e-mail.

**Older Adult & Caregiver Interview & Focus Group Guide**

Introduction Script (*accompanied by a Letter of Information & Consent Form*)

Thank you for taking the time to participate in this study. I want to start with a reminder that you may choose not to answer any of the questions and you can end your participation at any time during this interview. Your confidentiality will be respected and your name will not be associated with the data. With your permission, we will be audio-recording this session.

This project is about current processes older adults use to manage their healthcare with the help of their family members and how these processes can be improved or supported. I am specifically interested in how you keep track of information when you see your family doctor or other healthcare provider.

1. What types of personal health information, such as blood pressure, medications, etc. do you find useful to keep track of when going for your medical appointments?

*Probe: Mediation lists? Appointments?*

1. When you are in your medical appointment and your doctor or other healthcare provider shares information with you, what do you usually do with that information?

*Probe: do you write it down in a notebook or rely on memory?*

An app or application is a software program that you can use on tablets such as an iPad, or on mobile devices, such as a smart phone. Apps can have a variety of purposes and are sometimes used by people to keep track of things they think are important to them. A possible use is for recording and keeping track of health information. In our study, we are looking to find out whether older adults and their family members would benefit from using such an app, and what things older adults and family members may find useful. To begin this part of our conversation, I would like to go around the room and learn a bit about what kind of experiences you’ve had with mobile devices and apps. Could you each say a few words about that (go around the room so everyone has an opportunity to speak if they wish).

1. Do you feel that an app on your smartphone or tablet would be helpful for keeping track of the information that was discussed earlier? Why or why not?
2. What features would make you want to use the app? What would you want it to do?

*Probe: texting capabilities with a doctor, look at your own data, keep track of your own records, conversations, and appointments, reminders, service locator*

1. Would you be comfortable in using a tablet to keep track of your health information as part of a research study?
2. What are some concerns that would prevent you from using this type of app?

*Probe: Discomfort with using mobile devices? Use of technology? Privacy concerns? Low computer literacy? Not appropriate target population?*

1. Do you have any additional comments or feedback? *(index cards or can say in group)*

In getting ready for this meeting, I completed a literature search on existing apps that serve a similar purpose and would like your feedback on the results.

At the end of the interview, I will hand the participants the feature preference questionnaire and give them 5-10 minutes to complete and hand back to me.

**Field Testing Notes & Check-in Interview Questions**

1. Please write down your comments/thoughts about the **“Profile” section**

Point form notes are acceptable.

*(Some things to consider: How useful are the features in this section? Is it easy to navigate? Is it straightforward or confusing? What do you like about this section? What do you not like about this section? What else would you like us to know?)*

1. Please write down your comments/thoughts about the **“Care Team” section**

Point form notes are acceptable.

*(Some things to consider: How useful are the features in this section? Is it easy to navigate? Is it straightforward or confusing? What do you like about this section? What do you not like about this section? What else would you like us to know?)*

1. Please write your comments/thoughts about the **“Calendar” section**.

Point form notes are acceptable.

*(Some things to consider: How useful are the features in this section? Is it easy to navigate? Is it straightforward or confusing? What do you like about this section? What do you not like about this section? What else would you like us to know?)*

1. Please write down your comments/thoughts about the **“Health Tracker” section**

Point form and jot notes are acceptable.

*(Some things to consider: How useful are the features in this section? Is it easy to navigate? Is it straightforward or confusing? What do you like about this section? What do you not like about this section? What else would you like us to know?)*

1. Please write down your comments/thoughts about the **“Notification” section** Point form notes are acceptable.

*(Some things to consider: How useful are the features in this section? Is it easy to navigate? Is it straightforward or confusing? What do you like about this section? What do you not like about this section? What else would you like us to know?)*

1. Please write down your comments/thoughts about the **“Menu” section**.

Point form and jot notes are acceptable

*(Some things to consider: How useful are the features in this section? Is it easy to navigate? Is it straightforward or confusing? What do you like about this section? What do you not like about this section? What else would you like us to know?)*

**Field-Testing Check-In Questions:**

**Initial Conversation:**

1. Usability of the App
   1. Did you find it easy to navigate through the application? Why/Why not
   2. Was it easy to enter in personal information and health data? Why/Why not
   3. Did you have any problems or difficulties using the application?
      1. What features did you find challenging to use?
2. Suggestions for App Improvement
   1. What would you change about the app?
3. Personal Preferences
   1. What was your favourite feature on the app?
   2. What section of the app did you find most helpful?
4. Training/ Information Booklets
   1. Were the booklets helpful for using the app and the tablet?
   2. Was the in-app tutorial helpful?
   3. Was there any other information that you wished you had that you could not find in this booklet or in-app tutorial?
5. Final Comments and/or Suggestions?

**Follow-up Conversations:**

1. Usage since last check-in:
   1. Have you used the app to track information about your health or health care (Probe for conditions, medications, appointments)
   2. Have you used the app to prepare for or follow-up from any medical appointments?
   3. Which features or sections of the app have you found most useful?
2. Have you gotten more comfortable using the app? (If so, probe for what supported that)

1. Is there anything that I can help you with in the app?
2. Comments and suggestions for improvement of the app?
