# Supplementary material for: Lessons and Reflections From an Extended Co-design Process Developing an mHealth App With and for Older Adults: Multiphase, Mixed Methods Study
Source: JMIR Aging. 2022 Oct 28;5(4):e39189. doi: 10.2196/39189 (PMC9652733; doi:10.2196/39189)
Supplement: Multimedia Appendix 2 [file aging_v5i4e39189_app2.docx]

**Appendix B: Questionnaires**

**Feature Preference Questionnaire- Older Adults & Caregivers**

| Below is a list if of types of information and features that could be included in an “app” on a tablet or smartphone that patients or family members might use to keep track of their health information. On a scale of 1 to 5, 1 being not interested, 5 being very interested please rate each of the following statements. | | | | | | | | | | | |
| --- | --- | --- | --- | --- | --- | --- | --- | --- | --- | --- | --- |
| **Feature** | | **Scale** | | | | | | | | | |
|  |  | **Not**  **Interested** | | | | | **Very**  **Interested** | | | | |
| **My well-being** | | | | | | | | | | | |
| 1. I will have the ability to document my medication list and dosage | | 1 | | 2 | | 3 | | | 4 | | 5 |
| 1. I will have the ability to track my symptoms | | 1 | | 2 | | 3 | | | 4 | | 5 |
| 1. I will be able to create personal health goals | | 1 | | 2 | | 3 | | | 4 | | 5 |
| **I will be able to keep track of…** | | | | | | | | | | | |
| 1. My blood pressure | | 1 | | 2 | | 3 | | | 4 | | 5 |
| 1. My heart rate | | 1 | | 2 | | 3 | | | 4 | | 5 |
| 1. My immunization records | | 1 | | 2 | | 3 | | | 4 | | 5 |
| 1. My illnesses | | 1 | | 2 | | 3 | | | 4 | | 5 |
| 1. My exercise | | 1 | | 2 | | 3 | | | 4 | | 5 |
| 1. My friends and family involved in my care | | 1 | | 2 | | 3 | | | 4 | | 5 |
| 1. My glucose levels | | 1 | | 2 | | 3 | | | 4 | | 5 |
| 1. My oxygen levels | | 1 | | 2 | | 3 | | | 4 | | 5 |
| 1. My general well-being/feelings | | 1 | | 2 | | 3 | | | 4 | | 5 |
| 1. My health insurance information | | 1 | | 2 | | 3 | | | 4 | | 5 |
| **Some things I would like to see the app do…** | | | | | | | | | | | |
| 1. I can view a graph of my recorded symptoms | | 1 | | 2 | | 3 | | | 4 | | 5 |
| 1. I will have the ability to view, download and print an electronic file of health information | | 1 | | 2 | | 3 | | | 4 | | 5 |
| 1. I will be able to connect other devices to the app through Bluetooth (e.g. blood pressure monitor, scale) | | 1 | | 2 | | 3 | | | 4 | | 5 |
| 1. I will be alerted if my data falls out of target range (e.g. if my blood pressure is too high) | | 1 | | 2 | | 3 | | | 4 | | 5 |
| 1. I will be have the ability to give access to others (healthcare provider(s) or caregivers) | | 1 | | 2 | | 3 | | | 4 | | 5 |
| **Reminders to support my memory** | | | | | | | | | | | |
| 1. I will receive a pop-up reminder to take my medication(s) | | 1 | | 2 | | 3 | | | 4 | | 5 |
| 1. I will receive a pop up reminder to input my health data into the app | | 1 | | 2 | | 3 | | | 4 | | 5 |
| 1. I will receive a reminder to pop up when I choose   (E.g. Talk to my (doctor, nurse, specialist, etc.) about (blood pressure, blood sugar, etc.) | | 1 | | 2 | | 3 | | | 4 | | 5 |
| 1. I will receive e-mail notifications as a reminder (e.g. your cardiologist appointment is today at 12:00pm) | | 1 | | 2 | | 3 | | | 4 | | 5 |
| **My Appointments** | |  | |  | |  | | |  | |  |
| 1. I will have the ability to reschedule missed appointments | | 1 | | 2 | | 3 | | | 4 | | 5 |
| 1. I will have the option to rebook easily | | 1 | | 2 | | 3 | | | 4 | | 5 |
| 1. I will have the ability to track who created the appointment | | 1 | | 2 | | 3 | | | 4 | | 5 |
| How do I prepare for the appointment? | | | | | | | | | | | |
| 1. Wear certain clothes | | 1 | | 2 | | 3 | | | 4 | | 5 |
| 1. Limit eating or drinking | | 1 | | 2 | | 3 | | | 4 | | 5 |
| 1. Bring medications | | 1 | | 2 | | 3 | | | 4 | | 5 |
| 1. Bring health documentation | | 1 | | 2 | | 3 | | | 4 | | 5 |
| 1. Form(s) | | 1 | | 2 | | 3 | | | 4 | | 5 |
| 1. Others | | 1 | | 2 | | 3 | | | 4 | | 5 |
| I will be able to request feedback after my appointment has occurred such as…(32-36) | | | | | | | | | | | |
| 1. I like this service | | 1 | | 2 | | 3 | | | 4 | | 5 |
| 1. This service is helping me | | 1 | | 2 | | 3 | | | 4 | | 5 |
| 1. I don’t like this service | | 1 | | 2 | | 3 | | | 4 | | 5 |
| 1. I don’t think this service is helping me | | 1 | | 2 | | 3 | | | 4 | | 5 |
| 1. Option to enter in “other” reason | | 1 | | 2 | | 3 | | | 4 | | 5 |
| I will be able to record whether or not I attended the appointment and the reasons such as… (37-42) | | | | | | | | | | | |
| 1. Bad weather | | 1 | | 2 | | 3 | | | 4 | | 5 |
| 1. I didn’t have a way to get there | | 1 | | 2 | | 3 | | | 4 | | 5 |
| 1. I was not feeling well | | 1 | | 2 | | 3 | | | 4 | | 5 |
| 1. I had to take care of someone else | | 1 | | 2 | | 3 | | | 4 | | 5 |
| 1. It was closed | | 1 | | 2 | | 3 | | | 4 | | 5 |
| 1. Option to enter in “other” reason | | 1 | | 2 | | 3 | | | 4 | | 5 |
| I will be able to input information about the appointment such as…(43-50) | | | | | | | | | | | |
| 1. Appointment name (e.g. Cardiologist appointment, Dr. _________) | | 1 | | 2 | | 3 | | | 4 | | 5 |
| 1. Appointment type – (e.g. referral, service) | | 1 | | 2 | | 3 | | | 4 | | 5 |
| 1. Date and time | | 1 | | 2 | | 3 | | | 4 | | 5 |
| 1. Location (e.g. 123 King Street West, Grand River Hospital) | | 1 | | 2 | | 3 | | | 4 | | 5 |
| 1. Reason for visit (e.g. I am experiencing a lot of fatigue) | | 1 | | 2 | | 3 | | | 4 | | 5 |
| 1. Notes for parking (e.g. Parking available at the back) | | 1 | | 2 | | 3 | | | 4 | | 5 |
| 1. Notes for travel time (e.g. It takes 15 minutes to drive there) | | 1 | | 2 | | 3 | | | 4 | | 5 |
| 1. Important phone numbers (e.g. Phone number of the cardiologist is 123-456-7890) | | 1 | | 2 | | 3 | | | 4 | | 5 |
| **How I will communicate with people involved in my care** | | | | | | | | | | | |
| 1. I will be able to keep a contact list and information of all those involved in my care team (e.g. Doctor, Nurse, Specialists, etc.) | 1 | | 2 | | 3 | | | 4 | | 5 | |
| 1. I will have the option to send health data to my providers before appointments | 1 | | 2 | | 3 | | | 4 | | 5 | |
| 1. I will be able to give verbal instructions to the app instead of typing it | 1 | | 2 | | 3 | | | 4 | | 5 | |
| 1. Online communication with my providers, caregivers and other patients (e.g. live chat, texting or e-mail) | 1 | | 2 | | 3 | | | 4 | | 5 | |
| **Things that will help me use the app** | | | | | | | | | | | |
| 1. There will be a tutorial within the app to explain to me how to set-up and use it | 1 | | 2 | | 3 | | | 4 | | 5 | |
| 1. Having the option of a paper-based or hard copy version rather than online version | 1 | | 2 | | 3 | | | 4 | | 5 | |
| 1. I will be able to call a telephone support line if I need help using the app or setting it up | 1 | | 2 | | 3 | | | 4 | | 5 | |
| 1. I will be given a user manual with written instructions of how to use the app | 1 | | 2 | | 3 | | | 4 | | 5 | |
| 1. I will be given a face-to-face training session on how to use the app | 1 | | 2 | | 3 | | | 4 | | 5 | |
| 1. I will have access to an online user manual with instructions on how to use the app | 1 | | 2 | | 3 | | | 4 | | 5 | |
| **Additional Features (from participants)** | | | | | | | | | | | |
|  | | 1 | | 2 | | 3 | | | 4 | | 5 |
|  | | 1 | | 2 | | 3 | | | 4 | | 5 |
|  | | 1 | | 2 | | 3 | | | 4 | | 5 |
|  | | 1 | | 2 | | 3 | | | 4 | | 5 |
|  | | 1 | | 2 | | 3 | | | 4 | | 5 |

# Feature Preference Questionnaire- Healthcare Providers

| **Below is a list of types of information and features that could be included in an “app” on a tablet or smartphone that patients or family members might use to keep track of their health information. On a scale of 1 to 5, 1 being not interested, 5 being very interested please rate each of the following statements.** | | | | | | | | | | | |
| --- | --- | --- | --- | --- | --- | --- | --- | --- | --- | --- | --- |
| **Feature** | | **Scale** | | | | | | | | | |
|  |  | **Not**  **Interested** | | | | | **Very**  **Interested** | | | | |
| **Patient well-being** | | | | | | | | | | | |
| 1. Patients will have the ability to document their medication list and dosage | | 1 | | 2 | | 3 | | | 4 | | 5 |
| 1. Patients will have the ability to track their symptoms | | 1 | | 2 | | 3 | | | 4 | | 5 |
| 1. Patients will be able to create personal health goals | | 1 | | 2 | | 3 | | | 4 | | 5 |
| **Patients be able to keep track of…** | | | | | | | | | | | |
| 1. Blood pressure | | 1 | | 2 | | 3 | | | 4 | | 5 |
| 1. Heart rate | | 1 | | 2 | | 3 | | | 4 | | 5 |
| 1. Immunization records | | 1 | | 2 | | 3 | | | 4 | | 5 |
| 1. Illnesses | | 1 | | 2 | | 3 | | | 4 | | 5 |
| 1. Exercise | | 1 | | 2 | | 3 | | | 4 | | 5 |
| 1. Friends and family involved in their care | | 1 | | 2 | | 3 | | | 4 | | 5 |
| 1. Glucose levels | | 1 | | 2 | | 3 | | | 4 | | 5 |
| 1. Oxygen levels | | 1 | | 2 | | 3 | | | 4 | | 5 |
| 1. General well-being/feelings | | 1 | | 2 | | 3 | | | 4 | | 5 |
| 1. Health insurance information | | 1 | | 2 | | 3 | | | 4 | | 5 |
| **Some things I would like to see the app do…** | | | | | | | | | | | |
| 1. Patients can view a graph of their recorded symptoms | | 1 | | 2 | | 3 | | | 4 | | 5 |
| 1. Patients will have the ability to view, download and print an electronic file of health information | | 1 | | 2 | | 3 | | | 4 | | 5 |
| 1. Patients will be able to connect other devices to the app through Bluetooth (e.g. blood pressure monitor, scale) | | 1 | | 2 | | 3 | | | 4 | | 5 |
| 1. Patients will be alerted if their data falls out of target range (e.g., if my blood pressure is too high) | | 1 | | 2 | | 3 | | | 4 | | 5 |
| 1. Patients will be have the ability to give access to others (healthcare provider(s) or caregivers) | | 1 | | 2 | | 3 | | | 4 | | 5 |
| **Reminders to support their memory** | | | | | | | | | | | |
| 1. Patients will receive a pop-up reminder to take their medication(s) | | 1 | | 2 | | 3 | | | 4 | | 5 |
| 1. Patients will receive a pop up reminder to input their health data into the app | | 1 | | 2 | | 3 | | | 4 | | 5 |
| 1. Patients will receive a reminder to pop up when they choose   (e.g., Talk to my (doctor, nurse, specialist, etc.) about (blood pressure, blood sugar, etc.) | | 1 | | 2 | | 3 | | | 4 | | 5 |
| 1. Patients will receive e-mail notifications as a reminder (e.g. your cardiologist appointment is today at 12:00pm) | | 1 | | 2 | | 3 | | | 4 | | 5 |
| **My Appointments** | |  | |  | |  | | |  | |  |
| 1. Patients will have the ability to reschedule missed appointments | | 1 | | 2 | | 3 | | | 4 | | 5 |
| 1. Patients will have the option to rebook easily | | 1 | | 2 | | 3 | | | 4 | | 5 |
| 1. Patients will have the ability to track who created the appointment | | 1 | | 2 | | 3 | | | 4 | | 5 |
| How to prepare for their appointment? | | | | | | | | | | | |
| 1. Wear certain clothes | | 1 | | 2 | | 3 | | | 4 | | 5 |
| 1. Limit eating or drinking | | 1 | | 2 | | 3 | | | 4 | | 5 |
| 1. Bring medications | | 1 | | 2 | | 3 | | | 4 | | 5 |
| 1. Bring health documentation | | 1 | | 2 | | 3 | | | 4 | | 5 |
| 1. Form(s) | | 1 | | 2 | | 3 | | | 4 | | 5 |
| 1. Others | | 1 | | 2 | | 3 | | | 4 | | 5 |
| Patients will be able to request feedback after their appointment has occurred such as…(32-36) | | | | | | | | | | | |
| 1. I like this service | | 1 | | 2 | | 3 | | | 4 | | 5 |
| 1. This service is helping me | | 1 | | 2 | | 3 | | | 4 | | 5 |
| 1. I don’t like this service | | 1 | | 2 | | 3 | | | 4 | | 5 |
| 1. I don’t think this service is helping me | | 1 | | 2 | | 3 | | | 4 | | 5 |
| 1. Option to enter in “other” reason | | 1 | | 2 | | 3 | | | 4 | | 5 |
| Patients will be able to record whether or not they attended the appointment and the reasons such as… (37-42) | | | | | | | | | | | |
| 1. Bad weather | | 1 | | 2 | | 3 | | | 4 | | 5 |
| 1. They didn’t have a way to get there | | 1 | | 2 | | 3 | | | 4 | | 5 |
| 1. They were not feeling well | | 1 | | 2 | | 3 | | | 4 | | 5 |
| 1. They had to take care of someone else | | 1 | | 2 | | 3 | | | 4 | | 5 |
| 1. It was closed | | 1 | | 2 | | 3 | | | 4 | | 5 |
| 1. Option to enter in “other” reason | | 1 | | 2 | | 3 | | | 4 | | 5 |
| Patients will be able to input information about the appointment such as…(43-50) | | | | | | | | | | | |
| 1. Appointment name (e.g. Cardiologist appointment, Dr. _________) | | 1 | | 2 | | 3 | | | 4 | | 5 |
| 1. Appointment type – (e.g. referral, service) | | 1 | | 2 | | 3 | | | 4 | | 5 |
| 1. Date and time | |  | |  | |  | | |  | |  |
| 1. Location (e.g. 123 King Street West, Grand River Hospital) | | 1 | | 2 | | 3 | | | 4 | | 5 |
| 1. Reason for visit (e.g. I am experiencing a lot of fatigue) | | 1 | | 2 | | 3 | | | 4 | | 5 |
| 1. Notes for parking (e.g. Parking available at the back) | | 1 | | 2 | | 3 | | | 4 | | 5 |
| 1. Notes for travel time (e.g. It takes 15 minutes to drive there) | | 1 | | 2 | | 3 | | | 4 | | 5 |
| 1. Important phone numbers (e.g. Phone number of the cardiologist is 123-456-7890) | | 1 | | 2 | | 3 | | | 4 | | 5 |
| **How patients will communicate with people involved in their care** | | | | | | | | | | | |
| 1. Patients will be able to keep a contact list and information of all those involved in their care team (e.g. Doctor, Nurse, Specialists, etc.) | 1 | | 2 | | 3 | | | 4 | | 5 | |
| 1. Patients will have the option to send health data to their providers before appointments | 1 | | 2 | | 3 | | | 4 | | 5 | |
| 1. Patients will be able to give verbal instructions to the app instead of typing it | 1 | | 2 | | 3 | | | 4 | | 5 | |
| 1. Online communication with their providers, caregivers and other patients (e.g. live chat, texting or e-mail) | 1 | | 2 | | 3 | | | 4 | | 5 | |
| **Things that will help patients use the app** | | | | | | | | | | | |
| 1. There will be a tutorial within the app to explain to them how to set-up and use it | 1 | | 2 | | 3 | | | 4 | | 5 | |
| 1. Having the option of a paper-based or hard copy version rather than online version | 1 | | 2 | | 3 | | | 4 | | 5 | |
| 1. Patients will be able to call a telephone support line if they need help using the app or setting it up | 1 | | 2 | | 3 | | | 4 | | 5 | |
| 1. Patients will be given a user manual with written instructions of how to use the app | 1 | | 2 | | 3 | | | 4 | | 5 | |
| 1. Patients will be given a face-to-face training session on how to use the app | 1 | | 2 | | 3 | | | 4 | | 5 | |
| 1. Patients will have access to an online user manual with instructions on how to use the app | 1 | | 2 | | 3 | | | 4 | | 5 | |
| **Additional Features (from participants)** | | | | | | | | | | | |
|  | | 1 | | 2 | | 3 | | | 4 | | 5 |
|  | | 1 | | 2 | | 3 | | | 4 | | 5 |
|  | | 1 | | 2 | | 3 | | | 4 | | 5 |
|  | | 1 | | 2 | | 3 | | | 4 | | 5 |
|  | | 1 | | 2 | | 3 | | | 4 | | 5 |
